# Identification and Validation of VEGFR2 Kinase as a Target of Voacangine by a Systematic Combination of DARTS and MSI

**DOI:** 10.3390/biom10040508

**Published:** 2020-03-27

**Authors:** Yonghyo Kim, Yutaka Sugihara, Tae Young Kim, Sung Min Cho, Jin Young Kim, Ju Yeon Lee, Jong Shin Yoo, Doona Song, Gyoonhee Han, Melinda Rezeli, Charlotte Welinder, Roger Appelqvist, György Marko-Varga, Ho Jeong Kwon

**Affiliations:** 1 Chemical Genomics Global Research Lab., Department of Biotechnology, College of Life Science and Biotechnology, Yonsei University, Seoul 03722, Koreakty1273@yonsei.ac.kr (T.Y.K.); sungmincho@yonsei.ac.kr (S.M.C.); gyorgy.marko-varga@bme.lth.se (G.M.-V.); 2 Clinical Protein Science & Imaging, Department of Biomedical Engineering, Lund University, BMC D13, SE-221 84 Lund, Sweden; yutaka.sugihara@med.lu.se (Y.S.); melinda.rezeli@bme.lth.se (M.R.); charlotte.welinder@med.lu.se (C.W.); roger.appelqvist@bme.lth.se (R.A.); 3 Division of Oncology and Pathology, Department of Clinical Sciences Lund, Lund University, Barngatan 4, SE-221 85 Lund, Sweden; 4 Biomedical Omics Group, Korea Basic Science Institute, Ochang, Chungbuk 28119, Korea; jinyoung@kbsi.re.kr (J.Y.K.); jylee@kbsi.re.kr (J.Y.L.); jongshin@kbsi.re.kr (J.S.Y.); 5 Department of Biotechnology, College of Life Science and Biotechnology, Yonsei University, Seoul 03722, Korea; doona.s@yonsei.ac.kr (D.S.); gyoonhee@yonsei.ac.kr (G.H.); 6 Department of Surgery, Tokyo Medical University, 6-7-1 Nishishinjiku, Shinjuku-ku, Tokyo 160-0023, Japan; 7 Department of Internal Medicine, College of Medicine, Yonsei University, Seoul 03722, Korea

**Keywords:** target identification, target validation, label-free method for drugs, anti-angiogenesis, mechanism of action, receptor tyrosine kinases, curcumin, natural products

## Abstract

Although natural products are an important source of drugs and drug leads, identification and validation of their target proteins have proven difficult. Here, we report the development of a systematic strategy for target identification and validation employing drug affinity responsive target stability (DARTS) and mass spectrometry imaging (MSI) without modifying or labeling natural compounds. Through a validation step using curcumin, which targets aminopeptidase N (APN), we successfully standardized the systematic strategy. Using label-free voacangine, an antiangiogenic alkaloid molecule as the model natural compound, DARTS analysis revealed vascular endothelial growth factor receptor 2 (VEGFR2) as a target protein. Voacangine inhibits VEGFR2 kinase activity and its downstream signaling by binding to the kinase domain of VEGFR2, as was revealed by docking simulation. Through cell culture assays, voacangine was found to inhibit the growth of glioblastoma cells expressing high levels of VEGFR2. Specific localization of voacangine to tumor compartments in a glioblastoma xenograft mouse was revealed by MSI analysis. The overlap of histological images with the MSI signals for voacangine was intense in the tumor regions and showed colocalization of voacangine and VEGFR2 in the tumor tissues by immunofluorescence analysis of VEGFR2. The strategy employing DARTS and MSI to identify and validate the targets of a natural compound as demonstrated for voacangine in this study is expected to streamline the general approach of drug discovery and validation using other biomolecules including natural products.

## 1. Introduction

Identifying the protein targets of therapeutic natural products and deciphering the specific mechanisms of action at the molecular level are crucial steps in the development of natural products as drugs to treat human diseases [1,2]. Without the validation of targets and the cellular actions of natural products, these compounds may cause unexpected events, including adverse and toxic effects in patients. Thus, methods for the identification of protein targets and the understanding of molecular mechanisms of action of these natural products are pivotal for treating human diseases.

Given the importance and necessity of deciphering these parameters, there have been several technological attempts to successfully identify the protein targets for natural products [3,4]. Widely-applied approaches include affinity-based matrices that label or tag small molecules, such as affinity pull-downs and phage display methods [5,6,7]. Nevertheless, these methods have limitations, such as changes in structural properties may occur upon labeling with chemical probes, or upon tagging functional groups for immobilization; biological activities of natural products may change as a result of the alterations in the chemical structure; these processes incur a high cost, and are time and labor-consuming; and difficulty in modifying small molecules due to availability of only virtual three-dimensional structures [8,9]. To overcome these limitations, new strategies have been suggested for target identification and validation using label-free methods [6] with natural products. Specifically, the targets are validated by utilizing changes in thermodynamic properties and structural stability when a natural product directly interacts with a cognate protein target [5,10]. These methods apply thermal [11], proteolytic [12,13] or oxidative stress [14] to analyze changes in the structural stability of protein targets. One of these methods using proteolytic stress for target validation is the DARTS method. DARTS is based on the principle of increased stability of a protein target upon interaction with a natural product, which makes the complex less susceptible to proteolytic effects. The conformational changes induced upon the interaction between the natural product and the target protein thermodynamically stabilize the protein structure [12,13]. Moreover, through unbiased DARTS approaches in combination with mass spectrometry (MS) analysis, quantitative MS based-proteomics is utilized to identify multiple target proteins in drug-treated versus control samples [15].

Although these label-free methods have several advantages that overcome many of the difficulties associated with labeling methods, researchers are yet to identify and validate directly in vivo interactions between natural products and their target proteins at the tissue or cellular level. Recently, matrix-assisted laser desorption ionization-mass spectrometry imaging (MALDI-MSI) has emerged as a new technology to analyze the distribution of a natural product in tissues by directly measuring the molecular mass of said molecule from the tissue sections. MALDI-MSI has also been adapted to investigate the interactions between natural products and protein targets ex vivo. MALDI-MSI is an analytical mass spectrometry technology that identifies ion peaks from a natural product at 25–100 µm resolution [16,17]. This platform can be utilized to either detect all ion masses within a tissue microenvironment or to perform selected-ion monitoring (SIM) of a single, specific ion mass. Furthermore, with the automated computational procedures [18,19], the data generated by MALDI-MSI can exhibit the spatial localization of all the detected natural products as a single integrated image. Accordingly, MALDI-MSI is a powerful tool for validating the interactions between a natural product and its protein targets ex vivo without any labeling probes or chemical immobilization [20,21]. With respect to absorption, distribution, metabolism, and excretion (ADME), MALDI-MSI could prove to be an effective approach to provide valuable information about the in vivo effects of label-free compounds in patients [22,23].

Accordingly, for overcoming the conventional limitations of target identification and analyzing the localization of interaction between natural products and target proteins at the tissue level, we combined the aforementioned methods into a systematic procedure and applied the same for target validation. To validate DARTS-MSI as a successful systematic strategy for target identification of natural products, we applied the same for a target-validated compound, curcumin, as a positive control natural compound. In the previous studies, curcumin was shown to directly and irreversibly bind to aminopeptidase N (APN), which plays a key role in tumor angiogenesis and proliferation, inhibiting its activity and hence angiogenesis [24].

According to a previous report, we identified a small natural molecule with antiangiogenic activity [25]. This molecule is called voacangine and is extracted from *Voacanga africana*, *Trachelospermum jasminoides*, or *Tabernaemontana catharinensis*. Preliminary experiments suggested that voacangine potentially inhibits angiogenesis. This effect was observed in tube formation assays in endothelial cells (ECs) and vascularization of the chick chorioallantoic membrane. Additionally, we also reported that voacangine significantly inhibited VEGF-induced chemoinvasion activity on HUVECs in a dose-dependent manner. However, its mechanistic pathways and molecular targets are still uncovered and not fully understood. Therefore, we focused on the investigation for the mode of action in voacangine as the model natural compound by applying the aforementioned systematic approach and various molecular experiments. 

In the current study, we investigated the mode of action of voacangine via label-free DARTS and successfully identified VEGFR2 as a target protein responsible for the observed antiangiogenic properties of voacangine in ECs. The direct interaction between voacangine and VEGFR2 was validated in vivo in animal models and also by analyzing the localization of voacangine by MSI in xenograft tumor tissue sections. In addition, sunitinib, a marketed drug inhibiting tyrosine kinases by targeting not only VEGFR2 but also other RTKs (EGFR, PDGFR, and FGFR), was selected as a reference compound for comparing the potency on angiogenesis and tumor suppression with voacangine [26,27,28,29]. The strategy of employing DARTS and MSI to identify and validate the downstream targets of a natural compound as demonstrated for voacangine in this study can streamline the general process of drug discovery and validation of protein targets for other biomolecules including natural products. 

## 2. Materials and Methods 

### 2.1. Materials and General Methods

Curcumin (purity, ≥98%) was obtained from Sigma-Aldrich (St Louis, MO, USA). Voacangine (12-methoxyibogamine-18-carboxylic acid methylester) (purity, ≥98%) was purchased from THC Pharm (Frankfurt, Germany) [25], and the stock solution made in 100% dimethyl sulfoxide (DMSO) was stored at −20 °C, and diluted with the culture medium before the in vitro experiments. The working solution was freshly prepared in basal medium and the control group was treated with the same volume of DMSO as a vehicle control. Sunitinib (purity, ≥98%) was obtained from Sigma-Aldrich. Endothelial growth medium-2 (EGM-2) was purchased from Lonza (Walkersville, MD, USA). Dulbecco’’=s Modified Eagle Medium (DMEM), Roswell Park Memorial Institute (RPMI) 1640, and fetal bovine serum (FBS) were purchased from Invitrogen (Grand Island, NY, USA). Vascular endothelial growth factor (VEGF), Tumor Necrosis Factor-α (TNF-α), epidermal growth factor (EGF), basic fibroblast growth factor (bFGF), and platelet-derived growth factor-BB (PDGF-BB) were purchased from KOMA biotech (Seoul, Korea). The Transwell chamber system for chemoinvasion assay and Matrigel (growth factors reduced) were obtained from Corning Costar (Corning, NY, USA) and BD Biosciences (Bedford, MA, USA), respectively. Pronase and protease inhibitor cocktail tablets were obtained from Roche (Mannheim, Germany). Phosphatase inhibitor solution, Triton X-100, and dithiothreitol (DTT) were purchased from Sigma-Aldrich. Sodium chloride (NaCl), Tris, and glycine were obtained from Samchun Chemical Co., Ltd. (Seoul, Korea). Trifluoroacetic acid (TFA), high-performance liquid chromatography (HPLC)-grade methanol (≥99.8%) and the matrix compound, α-cyano-4-hydroxycinnamic acid (CHCA) were purchased from Sigma-Aldrich, and liquid chromatography–mass spectrometry (LC–MS) hypergrade acetonitrile (ACN) was obtained from Merck (Darmstadt, Germany). Primary antibodies of phospho-VEGFR2, VEGFR2, fibroblast growth factor receptor 1 (FGFR1), platelet-derived growth factor receptor α (PDGFRα), platelet-derived growth factor receptor β (PDGFR β), phospho- extracellular signal-regulated protein kinases 1 and 2 (ERK1/2), ERK1/2, phospho-protein kinase B (Akt), Akt, APN, and β-actin were purchased from Cell Signaling Technology (Beverly, MA, USA). Anti-β-III-tubulin was purchased from Millipore (Temecula, CA, USA). Anti-epidermal growth factor receptor 1 (EGFR1) and anti-voltage-dependent anion-selective channel 1 (VDAC1) were purchased from Abcam (Cambridge, UK). Anti-fibroblast growth factor receptor 5 (FGFR5) was purchased from Thermo Fischer Scientific (Waltham, MA, USA). Anti-cluster of differentiation 31 (CD31) was purchased from Novus (Littleton, CO, USA). Secondary antibodies of anti-rabbit immunoglobulin G (IgG) and anti-mouse IgG were purchased from Cell Signaling Technology. U87 glioblastoma cells (U87MG), human umbilical vein endothelial cells (HUVECs), and human hepatoma cell (HepG2) were purchased from Korea Cell Line Bank, Seoul, Korea.

### 2.2. In Vivo Mouse Tumor Xenograft Assays

Mice were housed in the pathogen-free facility of the Laboratory Animal Research Center in Yonsei University, Seoul, Korea. The mice were handled following the Institutional Animal Care and Use Committee (IACUC) (permission number: IACUC-A-201407-254-01, IACUC-A-201503-213-01, IACUC-A-201602-149-02, and IACUC-201603-422-01) and International Guidelines for the Ethical Use of Animals. U87MG cells (5 × 10^6^ cells) suspended in 200 μL phosphate-buffered saline (PBS)/Matrigel (1:1) were subcutaneously implanted into the dorsal flank of athymic nude mice (4-week-old female BALB/c nude mice, Orient Bio, Seoul, Korea). Once the tumors became palpable (50–100 mm^3^, ~2 weeks), mice were randomly selected and separated into four groups (6 mice per group), and intraperitoneally treated with vehicle, curcumin (60 mg/kg), and voacangine (10 mg/kg) daily. Sunitinib was administered orally (40 mg/kg) daily. Vehicle and drug solutions were prepared in saline:ethanol:Tween-80 (97.8:2:0.2). Tween-80 was used to enhance drug solubility. Tumor volume and mouse body weight were measured daily using the following formula: π/6 × length × width × height. Four hours after the last treatment (on day 12), mice were sacrificed, and tissue samples (tumors, livers, and kidneys) were obtained. The tissues were surgically removed and slowly frozen by placing tumors for 2 min on a plastic boat floating in a bath of isopentane that was supercooled with dry ice (−70 °C) [30]. All animal study protocols were performed following the Guidelines for Animal Experiments and were approved by the Department of Institutional Animal Care and Use Committee, Yonsei University, Seoul, Korea.

### 2.3. Growth Factor-Induced Chemoinvasion Assays

To determine the invasiveness of HUVECs in vitro, a Transwell chamber system with polycarbonate filter inserts containing 8.0 μm pores was used. The lower side of the filter was coated with 10 μL of gelatin (Sigma-Aldrich) (1 mg/mL), and the upper side was coated with 10 μL of Matrigel (growth factors reduced, 3 mg/mL in high-grade pure water). Voacangine was added to the lower chambers in the presence of the growth factors (VEGF, TNF-α, bFGF, PDGF-BB, and EGF, 30 ng/mL each), and HUVECs (FBS starvation for 17 h, 6 × 10^5^ cells/well) were placed in the upper chambers. The chambers were incubated at 37 °C for 16 h. The invasiveness of cells fixed with 70% methanol and stained with hematoxylin and eosin (H&E) was measured by counting the total number of cells on the lower side of the filter, using an Olympus IX70 microscope at 100× *g* magnification.

### 2.4. Drug Affinity Responsive Target Stability (DARTS) Assay

DARTS assay was performed as previously described [13,15]. Briefly, HUVECs were lysed using 0.5% Triton X-100 lysis buffer (50 mM Tris-HCl pH 7.5, 200 mM NaCl, 0.5% Triton X-100, 10% glycerin, 1 mM DTT) containing protease and phosphatase inhibitors. The supernatant from the cell lysates containing 2–3 mg/mL total protein was incubated with voacangine at the indicated concentrations at room temperature (RT) for 1 h, followed by proteolysis with pronase (1 µg/mL per sample) for 2, 5, and 10 min at RT. For curcumin treatment, the supernatant from the membrane fraction of HepG2 cell lysates containing 1.5 mg/mL total protein was incubated with curcumin at the indicated concentrations at RT for 3 h, followed by proteolysis with 10 µg/mL pronase per sample for 2, 5, and 10 min at RT. The final concentration of DMSO was 1% in all samples. To quench proteolysis, 6× sodium dodecyl sulfate (SDS) sample loading buffer (1 M Tris-hydrochloric acid (HCl) pH 6.8, SDS 10%, glycerol 60%, bromophenol blue 0.012%, and 0.6 M DTT) was added to each sample in a 1:3 ratio, thoroughly mixed, and boiled at 100 °C for 5 min. Samples were analyzed by immunoblotting with primary antibodies (APN, VDAC1, β-actin, VEGFR2, FGFR1, PDGFRα, and PDGFRβ) according to the manufacturer’s instructions.

### 2.5. In Silico Docking Simulation

All molecular docking analyses were performed with Discovery Studio 2016 software adopting the CHARMM (Chemistry at Harvard Macromolecular Mechanics) force field. The crystal structure of human VEGFR2 (Protein Data Bank code, 4AGD) was obtained from the Research Collaboratory for Structural Bioinformatics (RCSB) protein data bank. The protein structures of VEGFR2 were optimized by the Powell algorithm to minimized energy. To dock the ligands, the Ligandfit docking method was used. The parameters of Ligandfit were validated using the ligand from the VEGFR2 crystal structure. Voacangine was docked to the binding site of the protein and 10 poses were generated. The most predictive binding mode were determined based on various scoring functions (Ligscore1_Dreiding, Ligscore2_Dreiding, PLP1, PLP2, PMF, and DOCK_SCORE), and the binding energies were determined by calculating the binding energy of the most predictive binding mode.

### 2.6. Immunoblotting Analysis

Cell lysates were separated by 10% SDS–polyacrylamide gel electrophoresis (PAGE), and the proteins were transferred to polyvinylidene difluoride (PVDF) membranes using standard electroblotting procedures. Blots were blocked and immunolabeled overnight at 4 °C with primary antibodies. For immunoblotting of tumor samples, sections (10 µm thickness) from tumor tissues were collected and lysed in radioimmunoprecipitation assay (RIPA) buffer. Lysates from tumor samples were separated by 10% SDS-PAGE, and the proteins were transferred on PVDF membranes using standard electroblotting procedures. Blots were blocked and immunolabeled overnight at 4 °C with primary antibodies (phospho VEGFR2, VEGFR2 [31,32], phospho ERK, ERK, phospho Akt, Akt, and β3-tubulin) according to manufacturer’s instructions. Then membranes were washed with TBST (Tris-buffered saline with 0.05% Tween-20,) 3 times for 10 min each, and the secondary antibody was added and incubated for 1 h at RT. Immunolabeling was detected using an enhanced chemiluminescence (ECL) kit according to the manufacturer’s instructions.

### 2.7. Cell Growth Condition and Cell Proliferation Assays

Cell lines were grown according to the recommendations and protocols of the supplier. All cells were maintained at 37 °C in a humidified 5% CO_2_ incubator. All cells were seeded in 96-well plates at a density of 2000 cells/well. Voacangine was added to the cells to determine their effect on cell proliferation. Cells were grown for 72 h and growth was analyzed using the 3-(4,5-dimehylthiazol-2-yl)-2,5-diphenyl tetrazolium bromide (MTT) colorimetric assay.

### 2.8. Quantification of Microvessel Density

To measure the expression levels of the vascular marker CD31 in tumor sections, frozen sections were incubated with a primary anti-CD31 antibody. Frozen xenograft tumor sections (10 µm thickness) were incubated in primary antibody in 1% bovine serum albumin (BSA) overnight blocking buffer. After rinsing the primary antibody, the tumors were labeled with anti-rabbit Alexa-594 labeled secondary antibody (Invitrogen, 1:1000) for 1 h at RT and then counterstained with 4′,6-Diamidino-2-phenylindole (DAPI). Microvessel density was measured by counting the number of positive structures in three random fields. The images were obtained using a confocal laser scanning microscope LSM 700 (Carl Zeiss, Jena, Germany) from the whole tumor tissue at 400× *g* magnification.

### 2.9. Compound Detection and Analysis of Drug Distribution Using MSI

A MALDI LTQ Orbitrap XL mass spectrometer (Thermo Fisher Scientific, Waltham, MA, USA) was utilized for compound characterization, drug detection, and tissue imaging. For the matrix, 7.5 mg/mL α-CHCA was dissolved in 50% ACN and 50% Milli-Q water (high-grade pure water) containing 0.2% TFA. 

For tissue drug imaging, the freshly frozen tissues were cut into 10 μm sections using a cryotome (Thermo Fisher Scientific, Waltham, MA, USA) and placed on glass microscope slides (Superfrost ultra plus). After drying the tissue for 1 h at RT, 0.5 mL of the matrix solution was deposited stepwise onto the tissue by an airbrush. To control spraying conditions, the position of the airbrush was constantly maintained. Mass spectra were obtained using the Orbitrap mass analyzer (Thermo Fisher Scientific, Waltham, MA, USA) at 60,000 resolution (at *m*/*z* 400). Tissue sections were sampled in the 150−800 Da mass range in positive-ion mode with a 50 μm raster size. The nitrogen laser was operated at 10.0 μJ with activated automatic gain control. For MS/MS, the curcumin peak observed at *m*/*z* 369.14, and voacangine peak observed at *m*/*z* 369.21 were isolated with a 1.0 Da window, and fragmented at 40% normalized collision energy with a 30 ms activation time, 0.250 activation Q and the fragment ions were scanned at a normal scan rate in the linear ion-trap analyzer. The minimum signal required for MS/MS spectra generation was 500 counts. Spectra were analyzed with Xcalibur v 2.1.0. software. Visualization of the compound and fragment ions was performed with ImageQuest software (Version 1.0.1., Thermo Fisher Scientific, Waltham, MA, USA).

### 2.10. Quantitation of the Precursor Compound

For tissue quantitation, calibration curves of the drug and compounds were established in control tissue sections of the mice. Voacangine was diluted in 50% ACN containing 0.2% TFA. For each concentration, aliquots of 0.5 μL were applied to the tissue surface within the concentration range of 10 nM–1 mM. Spraying and detection conditions were identical to those used for the tissue sample analysis. The calibration curve was then used to estimate the tissue drug concentrations in in vivo-treated tumor sections.

### 2.11. Histochemical Analysis of Protein Target in Tissues and Compound Colocalization

To compare the immunofluorescence staining of the target protein and voacangine localization in tissues, frozen sections were sequentially cut from each tumor. Voacangine distribution was determined in the sections using MALDI-MSI and H&E staining. Sequential sections were labeled with anti-APN (1:100), anti-EGFR1 (1:50), anti-FGFR1 (1:50), anti-FGFR5 (1:50), and anti-VEGFR2 (1:50) [31]. The primary antibody incubation was followed by incubation with a fluorescent-tagged secondary antibody of anti-rabbit Alexa-488 (Invitrogen, 1:500). Nuclei were stained with DAPI (Invitrogen). The images were obtained using a confocal laser scanning microscope (LSM 700, Carl Zeiss) at a 200× *g* and 400× *g* magnification by title scanning the whole tumor tissue. Overlapping regions in the tumor tissue between compounds (curcumin and voacangine)-MSI and immunofluorescence staining of receptor tyrosine kinases (RTKs) were quantitated by Image J, Adobe Photoshop, and Qupath software [33] (0.2.0-m1) by counting the pixels of each merged region at identical image sizes.

### 2.12. Statistical Analysis

All data fitting and statistical analysis in different experimental groups are expressed as the mean ± standard deviation (S.D.) using GraphPad Prism and Microsoft Excel. The data shown in the study were obtained from at least three independent experiments. Statistical analyses were performed using an unpaired, two-tailed Student’s *t*-test. *P*-values less than 0.05 were considered statistically significant (* indicates *p* < 0.05, ** indicates *p* < 0.01, *** indicates *p* < 0.001).

## 3. Results and Discussions

### 3.1. Validation of the Systematic Combination of DARTS and MSI for Natural Product-Target Protein Interaction

**Figure 1:**
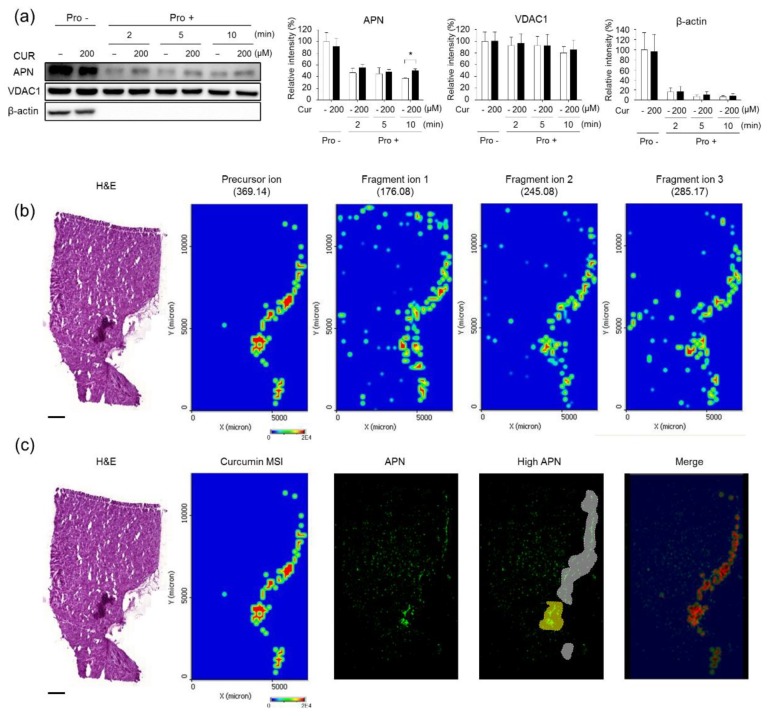
Validation of a systematic combination of drug affinity responsive target stability and mass spectrometry imaging (DARTS-MSI) for curcumin-aminopeptidase N (APN) interaction. (**a**) Analysis of direct binding of curcumin with APN in human umbilical vein endothelial cells (HUVECs) using the DARTS assay and immunoblotting. HUVEC lysates were incubated with curcumin and digested with pronase (0.1 μg/mL) at each incubation time. All images are the representative of three independent experiments. Each value represents the mean ± S.D. from three independent experiments. * *p* < 0.05 versus control. Cur: curcumin-treated, APN: aminopeptidase N. Pro -:Pronase non-treated; Pro +:Pronase treated; -:Non-treated (**b**) MALDI-MSI images of curcumin (precursor ion, fragment ion 1, fragment ion 2, and fragment ion 3) on curcumin-treated tumor tissue. The results shown are representative of three independent experiments. Scale bar, 1 mm. (**c**) Comparison of curcumin MSI (precursor ion) and immunofluorescence staining of the target protein, APN, in curcumin-treated tumor tissue. The transparent MSI image of curcumin is overlaid on the immunofluorescence staining image for APN and is visualized in the merged region (red). Scale bar, 1 mm.

Firstly, we performed a DARTS assay to validate the interaction between curcumin and APN (Figure 1a). The stability of APN significantly decreased after 2 min of treatment with pronase, but APN pretreated with curcumin before pronase treatment retained its stability. Secondly, we identified curcumin by mass spectra using MALDI-MS and MS/MS for the detection of curcumin at the tissue level (Figure S1a,b). Based on results obtained from mass spectra, quantitation of curcumin in tissue sections was conducted using MALDI-MSI. The precursor ion (curcumin, *m*/*z* 369.14) and fragment ions 1, 2, and 3 (*m*/*z* 176.08, *m*/*z* 245.08, and *m*/*z* 285.17, respectively) in the curcumin-treated tumor tissue sections were readily detected using MALDI-MSI (Figure 1b). In contrast, the precursor ion signals and fragment ions were not detected in control tissues (vehicle solution-treated tumors) (Figure S1c). Additionally, the intensity of curcumin was weaker in the liver and kidney tissue sections than in the tumor tissues of treated animals (Figure S1d). A merged image visualizing the transparent MSI signal of curcumin and the immunofluorescence image of APN was obtained (Figure 1c, red color). Notably, the highest concentrations of curcumin were observed in the tumor regions that expressed the highest concentrations of APN (yellow color in the high APN image). Through the quantitation of the merged pixel count, we observed that curcumin MSI showed high colocalization (72.65%) in the regions with the highest APN expression (Table 1). 

### 3.2. Identification of VEGFR2 as a Potent Target for Voacangine

**Figure 2:**
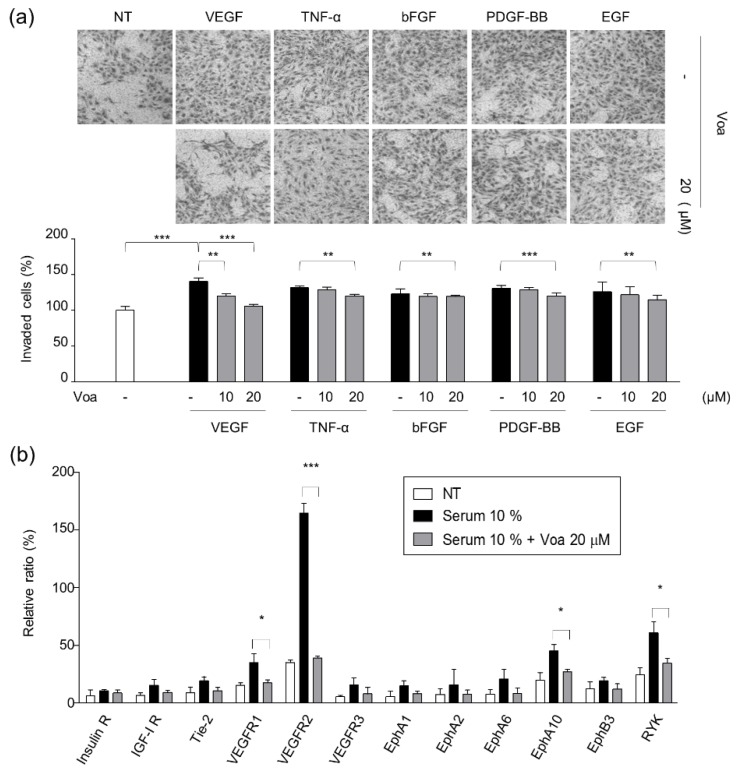
Voacangine specifically inhibited vascular endothelial growth factor (VEGF)-induced angiogenesis. (**a**) Effect of voacangine on chemoinvasion induced by various growth factors (VEGF, TNF-α, bFGF, PDGF-BB, and EGF). *** *p* < 0.001, ** *p* < 0.01 versus control of representative growth factors. -: Non-treated (**b**) Quantitation of the results from human p-RTKs array assay in HUVECs. The images are the representative of three independent experiments. Each value represents the mean ± S.D. from three independent experiments. *** *p* < 0.001, * *p* < 0.05 versus control. NT: non-treated control, Voa: voacangine-treated.

As the first step to determine the molecular mechanisms underlying the antiangiogenic effect and the signaling pathways involved in the process, the effect of voacangine on growth factor-induced chemoinvasion of ECs was investigated. HUVECs were treated with 10 and 20 μM voacangine in the presence of growth factors such as VEGF, TNF-α, bFGF, PDGF-BB, and EGF, and voacangine exhibited specific and potent suppression of VEGF-induced EC chemoinvasion (Figure 2a). In addition, a human phospho RTKs assay was also performed to further validate the inhibitory effect of voacangine on VEGF-mediated signaling activity (Figure 2b and Figure S2). The effect of voacangine on the phosphorylation of various RTKs was investigated in the HUVEC lysates. 

### 3.3. Validation of In Vitro Direct Interaction between Voacangine and VEGFR2 by DARTS

**Figure 3:**
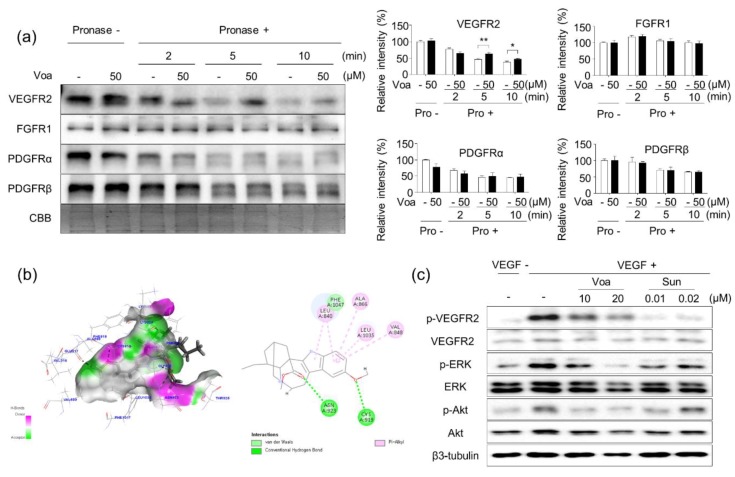
Voacangine specifically and directly binds to vascular endothelial growth factor receptor 2 (VEGFR2) (**a**) DARTS results from direct binding of voacangine to various receptor tyrosine kinases (RTKs) in HUVECs. HUVEC lysates were incubated with voacangine and digested with pronase (1 μg/mL) at each incubation time. Each value represents the mean ± S.D. from three independent experiments. ** *p* < 0.01 versus control, * *p* < 0.05 versus control. NT: non-treated control, Voa: voacangine-treated, Pro -:Pronase non-treated; Pro +:Pronase treated; -:Non-treated (**b**) In silico docking analysis using a 2D-diagram for validating the interaction between voacangine and VEGFR2 (juxtamembrane and kinase domains, RCSB Protein Data Bank number: 4AGD). Left panel, green (voacangine) is superimposed with VEGFR2 (grey). Right panel, binding motifs are illustrated with various interactions of voacangine with the ATP-binding pocket of VEGFR2. (**c**) Effect of voacangine on VEGF-induced VEGFR2 signaling. Protein levels were determined by immunoblotting using specific antibodies. The results shown are representative of three independent experiments. Voa: voacangine-treated; Sun: sunitinib-treated.

Next, DARTS was performed to explore whether voacangine directly binds to VEGFR2. As shown in Figure 3a, the stability of VEGFR2 significantly decreased after a 2 min pronase digestion. In contrast, VEGFR2 pretreated with voacangine before pronase treatment retained stability even after 5 and 10 min. 

To evaluate the binding of voacangine with VEGFR2, a docking model of human VEGFR2 based on its crystal structure (juxtamembrane and kinase domains, PDB number: 4AGD) was examined. In the virtual docking model of VEGFR2, two oxygen atoms of voacangine were found to reside in the hydrophobic pocket of VEGFR2 and form hydrogen bonds with Asn 923 and Cys 919, resulting in a high affinity and direct interaction with VEGFR2. The indole moiety of core directly interacted with the active residues in the VEGFR2 kinase domain (Leu 840, Val 848, Ala 866, and Leu 1035) via hydrophobic interactions (Pi-Alkyl interaction). The in-silico docking data suggested that voacangine directly interacts with VEGFR2 (Figure 3b). 

In pathological states, such as cancer development and tumor progression and other conditions exhibiting abnormal angiogenic phenotypes, growth factors such as VEGF are secreted from preexisting blood vessels to promote excessive cell growth. The secretion of VEGF by tumor cells ultimately leads to a remarkable promotion of angiogenesis [34,35]. Furthermore, VEGF-induced VEGFR2 signaling activates ERK and Akt by downstream phosphorylation of VEGFR2 kinase(s) and promotes angiogenesis by regulating the expression of the target gene, VEGF [36,37]. Accordingly, the effect of voacangine on VEGF-induced VEGFR2 activation and subsequent downstream signaling were investigated in HUVECs. Treatment with voacangine and sunitinib significantly suppressed the VEGF-induced phosphorylation of VEGFR2 and the downstream activation of ERK and Akt in a dose-dependent manner (Figure 3c). 

### 3.4. Voacangine Inhibits Xenograft Tumor Growth and Angiogenesis In Vivo

**Figure 4:**
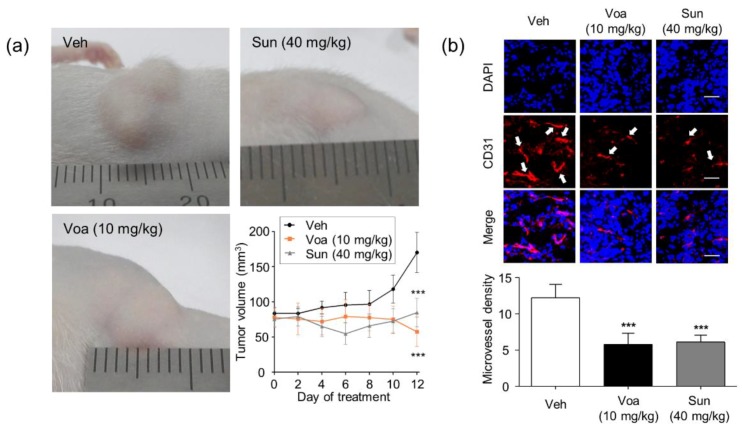
Voacangine inhibits xenograft tumor growth and angiogenesis in vivo. (**a**) Representative images of U87MG tumor xenograft on 12 days. Athymic nude mice bearing glioblastoma tumors consisting of U87MG glioblastoma cells were treated with vehicle, voacangine (10 mg/kg, intraperitoneal treatment), or sunitinib (40 mg/kg, oral treatment). *** *p* < 0.001 versus vehicle treatment. (**b**) Effect of voacangine or sunitinib treatment on the expression levels of the vascular marker, CD31 in tumor tissues. All images shown are representative of three independent experiments. White arrows indicate CD31 expression. Original magnification of fluorescence images for CD31 staining: 400× *g*. Scale bar, 50 μm. Microvessel density was measured by counting the number of CD31-positive structures in three random fields. Each value represents the mean ± S.D. from three independent experiments. *** *p* < 0.001 versus vehicle treatment. Veh, vehicle-treated; Voa, voacangine-treated; Sun, sunitinib-treated.

Validation of voacangine as a new VEGFR2-targeting antiangiogenic and antitumor compound was achieved by investigating its effect on tumor growth in the U87MG cell glioblastoma xenograft mouse model. U87MG glioblastoma cells form aggressive angiogenic solid tumors that exhibit high levels of VEGF and VEGFR2 [38]. As a reference, data were compared with those for sunitinib (SU11248, Sutent), a known VEGFR2-targeting anticancer drug. Both voacangine and sunitinib significantly inhibited tumor growth (Figure 4a) in 6 to 12 days without causing overt toxicity, as no significant weight loss was observed in the mice (Figure S4). As shown in Figure 4b, the expression levels of the blood vessel marker, CD31, were significantly lower in tumor-bearing mice treated with either voacangine (47.3%) or sunitinib (50%) than that of control.

High-resolution histological inspection revealed a cellular presentation within these tumors that showed a high degree of heterogeneity in cell size and shape. The sunitinib dosage (40 mg/kg, oral treatment) administered in these studies was selected from the dosage administered in the previous mice experiments [29,39,40]. Voacangine at a dosage of 10 mg/kg was administered at 48-h intervals for 14 days in the disease mouse model. A similar tendency of stabilized tumor growth was observed in mice treated with both, voacangine and sunitinib, with a fast growth onset that began on day 5, and then considerably expanded by day 8. These xenograft studies were performed with 6 animals per group, which showed a high degree of tumor growth consistency. 

**Figure 5:**
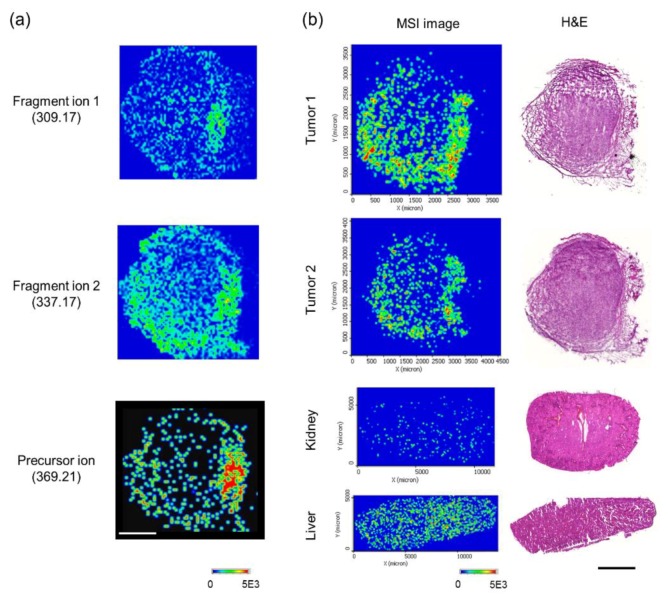
Identification of voacangine in tissue sections using mass spectrometry imaging (MSI). (**a**) MALDI-MSI signal for voacangine (precursor ion, fragment ion 1, and fragment ion 2) from voacangine-treated tumor tissue. The results shown are representative of three independent experiments. Scale bar, 1 mm. (**b**) Comparison of voacangine MSI (the precursor ion) between other voacangine-treated tumors and organ tissues (kidney and liver).

Next, the identification of voacangine in vivo by MSI was conducted. The precursor ion (voacangine, *m*/*z* 369.2162) (Figure S5a) fragment ions 1 and 2 (*m*/*z* 309.17 and *m*/*z* 337.17, respectively) (Figure S5b) in voacangine-treated tumor tissue sections were readily-detected using MALDI-MSI (Figure 5a). In contrast, the precursor compound signal and fragment ions for voacangine were not detected in the vehicle solution-treated tumor tissues (Figure S7). Additionally, in the liver and kidney tissue sections of treated animals, the voacangine signal was detected with weaker intensity than that in the tumor tissue (Figure 5b).

### 3.5. Validation of Target Interaction by Colocalization of Voacangine with VEGFR2 and Other RTKs

**Figure 6:**
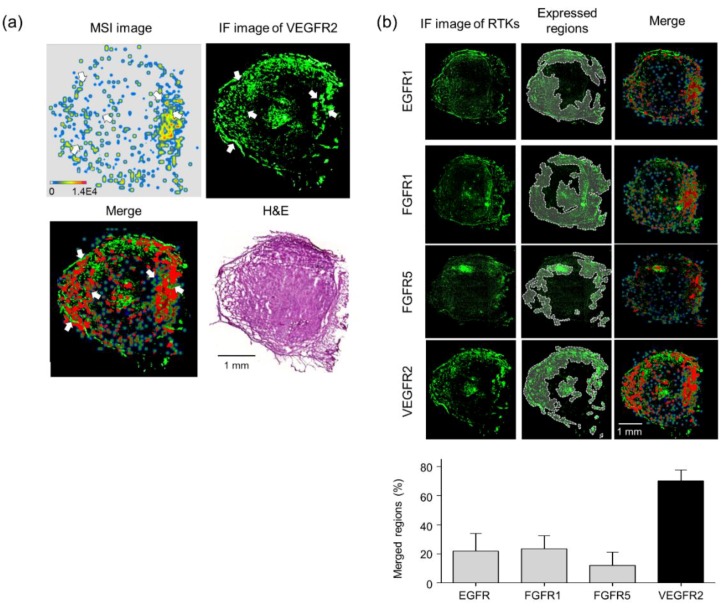
Validation of target interaction by colocalization of voacangine with VEGFR2 and other RTKs. (**a**) Comparison of voacangine MSI (precursor ion) and immunofluorescence staining of the target candidate, VEGFR2, in voacangine-treated tumor tissue. The overlaid image with transparent MSI signal on the immunofluorescence staining image for VEGFR2 is visualized in the merged region (red). Scale bar, 1 mm. (**b**) Comparisons and quantitation of merged regions for voacangine distribution and RTK receptors (EGFR1, FGFR1, FGFR5, and VEGFR2) in tumor tissues. Regions of expression for each RTK are indicated with white dashed lines on the immunofluorescence images. The merged regions are visualized in red. The results shown are representative of three independent experiments. Each value represents the mean ± S.D. from three independent experiments. Scale bar, 1 mm.

As shown in Figure S8, each tumor-bearing mouse showed high VEGFR2 expression, indicating that the tumor-bearing tissues also expressed high levels of VEGFR2 which were significantly reduced by voacangine treatment. From these observations, an overlay image was visualized with a transparent MSI signal of voacangine and the immunofluorescence image of VEGFR2 in a merged region (Figure 6a, red color). Next, immunofluorescence was performed to analyze the interaction between voacangine and various other RTKs (EGFR1, FGFR1, FGFR5, and VEGFR2) in voacangine-treated tumor tissues (Figure 6b) by quantifying the pixel counts from the merged regions (Table 2). The overlaid transparent MSI image was quantitated with the immunofluorescence images of various RTKs. The merged regions are highlighted in red. 

### 3.6. Discussions

Natural products have been widely used as pharmacological or nutraceutical agents for effectively treating various human diseases due to their significant biological activities with diverse chemical structures. Given the advantages of natural products, there have been many obstacles to address the exact mode of action and in vivo effects on possible target proteins. Most often these problems arise due to difficulties in chemical modification of these natural products and alteration in the chemical and biological properties during these processes. 

In this study, we attempted to overcome these hurdles by developing a new systematic procedure to identify targets of label-free natural products in vitro and in vivo with DARTS and MALDI-MSI, respectively. In Figure 1 and Table 1, curcumin pretreatment significantly protected APN digestion from pronase even at 10 min (*p* = 0.0185); but the digestion of other proteins, VDAC1 and β-actin remained unaffected, suggesting that curcumin specifically binds to APN, as reported by the earlier studies [24]. Further, these results strongly suggested that there is a stronger curcumin-binding and localization in the tumor tissue with elevated levels of APN expression. From these observations with curcumin, we suggest that the combination of DARTS and MALDI-MSI could be used as a systematic procedure for validation of in vivo target interaction with label-free natural products.

After validating the interaction of curcumin with APN as a proof of concept case, we utilized this approach for voacangine, a natural antiangiogenic compound, without any known targets. As shown in Figure 2a, HUVECs were treated with 10 and 20 μM voacangine in the presence of growth factors such as VEGF, TNF-α, bFGF, PDGF-BB, and EGF, and voacangine exhibited specific and potent suppression of VEGF-induced EC chemoinvasion. These results demonstrated that voacangine did not show inhibitory activity against the other growth factors but specifically inhibit VEGF-mediated signaling. In VEGF-induced conditions, voacangine specifically suppressed the phosphorylation of VEGFR2. This was further validated by the p-RTK array assay, wherein the phosphorylation profiles of various RTKs were analyzed. Furthermore, from the 12 RTKs activated by serum out of the 45 RTKs, voacangine specifically inhibited the VEGF-induced phosphorylation of VEGFR2 (Figure 2b, Figure S2). Accordingly, the following experiments on the activity of voacangine focused on VEGFR2.

Using DARTS technology, VEGFR2 was identified as a cellular target protein of voacangine. Notably, pretreatment with voacangine resulted in limited VEGFR2 digestion upon pronase treatment; however, the digestion of other RTKs, FGFR1, PDGFRα, and PDGFRβ remained unaffected, suggesting that voacangine specifically binds to VEGFR2, but not other RTKs (Figure 3a). The in vitro inhibitory activity of voacangine significantly inhibited the phosphorylation of VEGFR2 and downstream signaling proteins, such as ERK and Akt, in HUVECs (Figure 3c). These results demonstrated that the antiangiogenic mechanisms of voacangine action affect the VEGFR2 mediated signaling pathway. These also have been well-known that VEGF-mediated signaling plays a key role in tumor angiogenesis [34,35,41], and secreted VEGF binds to VEGFR2 that is expressed on the vascular endothelium. Subsequently, an angiogenic response is evoked that leads to the activation of VEGFR2 signaling [41]. VEGF-stimulated VEGFR2 induces the phosphorylation of downstream signaling kinases, including ERK and Akt which promote migration, proliferation, invasion, adhesion, and tube formation in ECs [36,37]. Therefore, targeting VEGFR2 is considered a promising strategy and an important therapeutic approach for treating diseases associated with angiogenesis, such as cancer [42].

Further evidence revealed that the effects of voacangine significantly correlated with the levels of VEGFR2 expression in different cell lines. The expression of VEGFR2 in HUVEC, U87MG, and Panc-1 cells was higher than that in the remaining cell lines (Figure S3a). As shown in Figure S3b, voacangine significantly inhibited cell proliferation in HUVEC, U87MG, and Panc-1 cells (Table S1). From the examination of the effects of voacangine on several cell lines with different VEGFR2 expression levels demonstrated that voacangine exerted its biological effects by specifically inhibiting proliferation in cells with high levels of VEGFR2 expression. Further, these results suggested that voacangine could be used as a specific inhibitor targeting VEGFR2-overexpressing cells (Figure S3). 

In the U87MG xenograft tumor mouse model, where VEGFR2 is highly expressed, voacangine significantly suppressed tumor growth and microvessel density in vivo without significant toxicity (Figure S4). The outcome of voacangine treatment suggested that it reduced the tumor growth to a basal level (as determined in the control groups), similar to treatment with sunitinib, which returned the tumors to baseline levels during the 14 days (Figure 4). Notably, this observation suggests that voacangine may cause dual inhibition of tumor angiogenesis and tumor growth, thus resulting in a potent antitumor activity. These results also demonstrated that voacangine might be a promising candidate for effective treatment of aggressive glioblastomas which are resistant to various chemotherapies [43]. Additionally, these data are in accord with the effects of the known VEGFR inhibitor, sunitinib. Based on these results, voacangine inhibited angiogenesis and tumor proliferation in vivo by directly targeting VEGFR2-overexpressing cancer cells, similar to sunitinib. 

To further validate VEGFR2 as the target receptor of voacangine, the interaction was analyzed in vivo. We established a novel systematic combination to compare the localization of small molecules and their protein targets using MALDI-MSI and immunofluorescent staining. From the MALDI-MS analyses, voacangine was identified at *m*/*z* 369.21, as well as its two major fragment ions were identified (Figure S5). On the surface of the tissue sections, a single droplet of voacangine could be detected by MALDI-MSI (Figure S6). The concentration of the voacangine and the precursor ion signal intensity linearly correlated from 0.01–100 μM. Voacangine precursor ions and its fragment ions were detected and colocalized with the tumor tissue from voacangine-treated mice. Furthermore, voacangine precursor ions and its fragment ions were not detected in control tissues, confirming that voacangine and the fragment ions observed by MALDI-MSI did not originate from the matrix or tissue. A comparative analysis of MALDI-MSI of voacangine ions and immunofluorescence images of other RTKs (EGFR1, FGFR1, FGFR5, PDGFRα, and PDGFβ), revealed that the highest concentrations of voacangine were observed in the tumor regions that expressed the highest concentrations of VEGFR2. This suggested a stronger voacangine-binding and localization in tumor tissues with elevated VEGFR2 expression (Figure 6).

In a previous study, high concentrations of sunitinib colocalized with high expression levels of VEGFR2 [31]. Akin to the marketed drug sunitinib, these results demonstrated that voacangine directly interacts with VEGFR2 and therefore, can potentially be considered as a promising natural compound for suppressing angiogenesis and tumor growth by targeting VEGFR2. Among VEGFR2-targeting drugs, sunitinib is the most widely used drug for treating cancer patients [26]. Sunitinib is a multi-targeting drug and a receptor tyrosine kinase inhibitor that inhibits signaling via key angiogenic receptors, including VEGFRs, PDGFRs, and FGFRs [27]. In this study, the newly identified antiangiogenic small molecule, voacangine, was shown to interact specifically with VEGFR2. Voacangine treatment resulted in a decrease in VEGFR2 kinase activity in vitro (Figure 2b and Figure S2) and reduced its expression levels in vivo (Figure S8).

Recently, many reports have demonstrated that sunitinib directly targets VEGFR2 to inhibit cancer progression in patients [44,45]. It is specifically administered as a first-line treatment to patients with advanced renal cell carcinoma (RCC) and imatinib-resistant gastrointestinal stromal tumors [46,47]. Despite the significant benefits of sunitinib treatment related to progression-free survival and disease stabilization in patients, almost all patients acquire resistance to sunitinib and relapse [48,49]. Approximately, 70% of patients show an initial response, while the remaining 30% show primary (intrinsic) resistance. Furthermore, 70% of patients acquired extrinsic resistance within 6–15 months. To treat patients with sunitinib resistance, the development of new VEGFR2 inhibitors with distinct structures and pharmacological activities is imperative for improved cancer therapy. As a possible drug candidate targeting VEGFR2 kinase, voacangine significantly inhibited in vitro and in vivo angiogenesis by directly and specifically interacting with VEGFR2. Hence, further development of voacangine as a new scaffold compound targeting the VEGFR2 kinase could provide a new option to treat cancer patients with resistance to sunitinib. Additionally, identifying and developing drug replacements from natural products such as voacangine will potentially reduce unpredicted and adverse side effects, and provide a promising strategy to improve the efficiency of small molecules in preclinical and clinical stages. 

## 4. Conclusions

**Figure 7:**
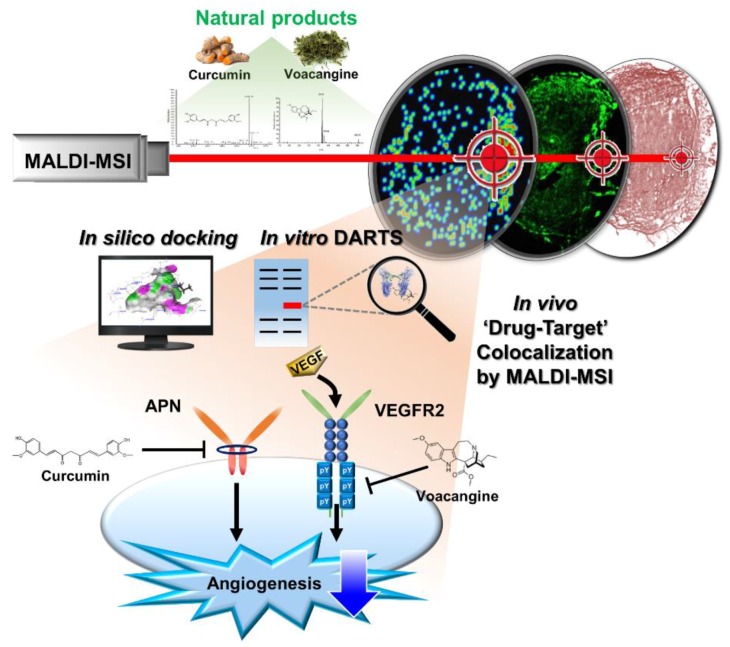
Summary of the study. The systematic approach using the combination of DARTS-MSI for in vitro and in vivo target identification and validation of natural products.

Here, we demonstrate an effective and novel systematic combination method, consisting of a label-free method for target identification of natural products and their in vivo validation with information on “on-target” effects and bioimaging data consisting of molecular interactions in tissue samples (Figure 7). This combinatorial technique is effective not only for voacangine but could also be effectively used for many tricky natural products and could boost target identification and hence, drug development. This study provides a new systematic approach to overcome many of the problems associated with currently available methods used for in vitro and in vivo target identification and validation. Our study represents a new means to identify and validate protein targets of natural compounds as “cold compounds” and eases the exploration of the mode of action of these natural products in vitro and in vivo without any chemical modifications. These results also provide new insights into the evaluation of drug actions in tissues and the colocalization of drugs and their respective targets in vivo.

## Figures and Tables

**Table 1:** Comparison with the highest concentration of curcumin and the highest APN expressed regions in the tumor regions. Each value represents the mean from three independent experiments.

Quantitation	Pixels	Merged Regions/Cur-MSI (%)
MALDI-MSI (curcumin)	44,408	100
Merged regions (high curcumin and high APN)	32,264	72.65

**Table 2:** Comparison with other RTKs in voacangine-treated tissues. Each value represents the mean from three independent experiments. IF: immunofluorescence-stained regions. Voa, voacangine.

Quantitation		Pixels	IF/Voa-MSI (%)
MALDI-MSI (voacangine)		71,406	100
Merged regions (red)	EGFR1	15,572	21
	FGFR1	16,745	23
	FGFR5	8616	12
	VEGFR2	50,039	70

## Supplementary Materials

The following are available online at https://www.mdpi.com/2218-273X/10/4/508/s1, Figure S1: Characterization of curcumin by MALDI-MS, MS/MS, and MSI, Figure S2: Voacangine specifically inhibits VEGFR2 signaling, Figure S3: Effects of voacangine on tumor growth and angiogenesis-related to VEGFR2 expression levels, Figure S4: Body weight of in vivo xenograft model, Figure S5: Characterization of curcumin by MALDI-MS and MS/MS, Figure S6: Quantitation of a droplet of voacangine on tumor tissue sections, Figure S7: Comparison of voacangine MSI between vehicle-treated tumor tissue, Figure S8: Effect of voacangine on the expression of VEGFR2, Table S1: IC50 values of voacangine in study cell lines.
