# Supplementary material for: Identification and Validation of VEGFR2 Kinase as a Target of Voacangine by a Systematic Combination of DARTS and MSI
Source: Biomolecules. 2020 Mar 27;10(4):508. doi: 10.3390/biom10040508 (PMC7226133; doi:10.3390/biom10040508)
Supplement: Supplementary file 1 [file biomolecules-10-00508-s001.pdf]

*Supplementary Materials*

# Identification and validation of VEGFR2 kinase as a target of voacangine by a systematic combination of DARTS and MSI

**Yonghyo Kim[1,2,3], Yutaka Sugihara[2,3], Tae Young Kim[1], Sung Min Cho[1], Jin Young Kim[4], Ju Yeon Lee[4], Jong Shin Yoo[4], Doona Song[5], Gyoonhee Han[5], Melinda Rezeli[2], Charlotte Welinder[2,3], Roger Appelqvist[2], György Marko-Varga[1,2,6], and Ho Jeong Kwon[1,7,*]**

[1] Chemical Genomics Global Research Lab., Department of Biotechnology, College of Life Science and Biotechnology, Yonsei University, Seoul 03722, Republic of Korea; yonghyo.kim@med.lu.se (Y.K.); kty1273@yonsei.ac.kr (T.Y.K.); sungmincho@yonsei.ac.kr (S.M.C.)

[2] Clinical Protein Science & Imaging, Department of Biomedical Engineering, Lund University, BMC D13, Lund SE-221 84, Sweden; yutaka.sugihara@med.lu.se (Y.S.); melinda.rezeli@bme.lth.se (M.R.); charlotte.welinder@med.lu.se (C.W.); roger.appelqvist@bme.lth.se (R.A.); gyorgy.marko-varga@bme.lth.se (G.M.-V.)

[3] Division of Oncology and Pathology, Department of Clinical Sciences Lund, Lund University, Barngatan 4, Lund SE-221 85, Sweden.

[4] Biomedical Omics Group, Korea Basic Science Institute, Ochang, Chungbuk 28119, Republic of Korea; jinyoung@kbsi.re.kr (J.Y.K.); jylee@kbsi.re.kr (J.Y.L.); jongshin@kbsi.re.kr (J.S.Y.)

[5] Department of Biotechnology, College of Life Science and Biotechnology, Yonsei University, Seoul 03722, Republic of Korea; doona.s@yonsei.ac.kr (D.S.), gyoonhee@yonsei.ac.kr (G.H.)

[6] Department of Surgery, Tokyo Medical University, 6-7-1 Nishishinjiku, Shinjuku-ku, Tokyo 160-0023, Japan.

[7] Department of Internal Medicine, College of Medicine, Yonsei University, Seoul 03722, Republic of Korea.

* Correspondence: kwonhj@yonsei.ac.kr (H.J.K.); Tel.: +82-2-2123-5883

---

**Supplementary materials and methods**

**Figure S1.** Characterization of curcumin by MALDI-MS, MS/MS, and MSI.

**Figure S2.** Voacangine specifically inhibits VEGFR2 signaling.

**Figure S3.** Effects of voacangine on tumor growth and angiogenesis related to VEGFR2 expression levels.

**Figure S4.** Body weight of in vivo xenograft model.

**Figure S5.** Characterization of curcumin by MALDI-MS and MS/MS.

**Figure S6.** Quantitation of a droplet of voacangine on tumor tissue sections.

**Figure S7.** Comparison of voacangine MSI between vehicle-treated tumor tissue.

**Figure S8.** Effect of voacangine on the expression of VEGFR2.

**Table S1.** $IC_{50}$ values of voacangine in study cell lines.

## Supplementary materials and methods

*Characterization of curcumin by MALDI-MS, MS/MS, and MSI.*

We identified curcumin by mass spectra using MALDI-MS and MS/MS for detection of curcumin at the tissue level. A droplet of curcumin mixed with the matrix solution $\alpha$-cyano-4-hydroxycinnamic acid ($\alpha$-CHCA, 7.5 mg/mL) was analyzed by MALDI-MS (Figure S1a). Intact curcumin was detected at *m/z* 369.14, and the three major fragment ions of the molecule were observed in the MS/MS data at *m/z* 176.08, *m/z* 245.08, and *m/z* 285.17, respectively (Figure S1b).

*Human phosphoreceptor tyrosine kinases (p-RTKs) array assay.*

Human Phospho-Receptor Tyrosine Kinase Array Kit was used to determine the effect of voacangine on relative levels of tyrosine phosphorylation of 49 RTKs, according to the manufacturer's protocol (Figure S2). Briefly, cells were lysed in lysis buffer from the kit containing protease inhibitors. The arrays were incubated in 500 μg total cell lysate overnight at 4 °C after blocking for 1 h with array buffer 1 from the kit. Protein concentration was determined using Bradford protein assay and was adjusted to ensure equal protein quantities. The arrays were washed and incubated with an anti-phospho-tyrosine-HRP (horseradish peroxidase) antibody (1:5000). Signal was detected using a Clarity Western ECL Substrate, according to the manufacturer's instructions, and signals were captured and quantitated using a ChemiDoc chemiluminescence imaging system. The relative intensities of the averaged signal from each pair of duplicated spots were determined as a ratio relative to the negative control spots.

*The effect of voacangine on cell lines with various VEGFR2 expression levels.*

Human umbilical vein endothelial cells (HUVECs, passages 4–8), cervical cancer (HeLa), non-small cell lung cancer cells (A549), colon cancer cells (HCT116), liver hepatocellular cancer cells (HepG2), breast cancer cells (MCF7 and MDA-MB-231), pancreatic cancer cells (Panc-1), U87 glioblastoma cells (U87MG), and prostate cancer cells (PC3) were purchased from Korea Cell Line Bank, Seoul, Republic of Korea. Gastric cancer cells (YCC16) were provided from Yonsei Medical School, Seoul, Republic of Korea. These cells were grown according to the recommendations and protocols of the supplier. All cells were maintained at 37°C in a humidified 5% CO2 incubator. To validate the biological relevance of VEGFR2 as a target protein of voacangine, the effect on the expression levels of VEGFR2 in a range of cell lines was investigated. One non-cancerous cell line (HUVEC) and 9 cancer cell lines were used. These were non-small cell lung cancer (A549), colon cancer (HCT116), liver hepatocellular cancer (HepG2), breast cancer (MCF7 and MDA-MB-231), pancreatic cancer (Panc-1), glioblastoma (U87MG), cervical cancer (HeLa), prostate cancer (PC3), and gastric cancer (YCC16) cells. The basal expression levels of VEGFR2 in the 10 cell lines were assessed by immunoblotting (Figure S3a). The cell lines were grown for 72 h in the presence of voacangine at concentrations ranging from 0–50 μM and the cell growth was analyzed using MTT assays (Figure S3b).

*Characterization of voacangine by MALDI-MS and MS/MS analysis.*

To address whether the antitumor activity of voacangine against glioblastoma xenografts originated from the interaction between the voacangine and the identified target protein, VEGFR2, the monoisotopic mass of voacangine was firstly identified by MALDI-MS. A droplet of voacangine mixed with the matrix solution $\alpha$-cyano-4-hydroxycinnamic acid ($\alpha$-CHCA, 7.5 mg/mL) was analyzed by MALDI-MS (Figure S5a). Intact voacangine was detected at *m/z* 369.21 and two major fragment ions of the molecule were observed in the MS/MS data at *m/z* 309.17, and *m/z* 337.17 (Figure S5b).

*Quantitation of voacangine in tumor tissue samples using MSI.*

Quantitation of voacangine in tissue sections was conducted using MALDI-MSI (Figure S6a). Quantitation of voacangine in tissue sections exhibited a linear correlation between concentration and signal intensity. As shown in Figure S6b, calibration curves of voacangine in tumor tissue samples were obtained.

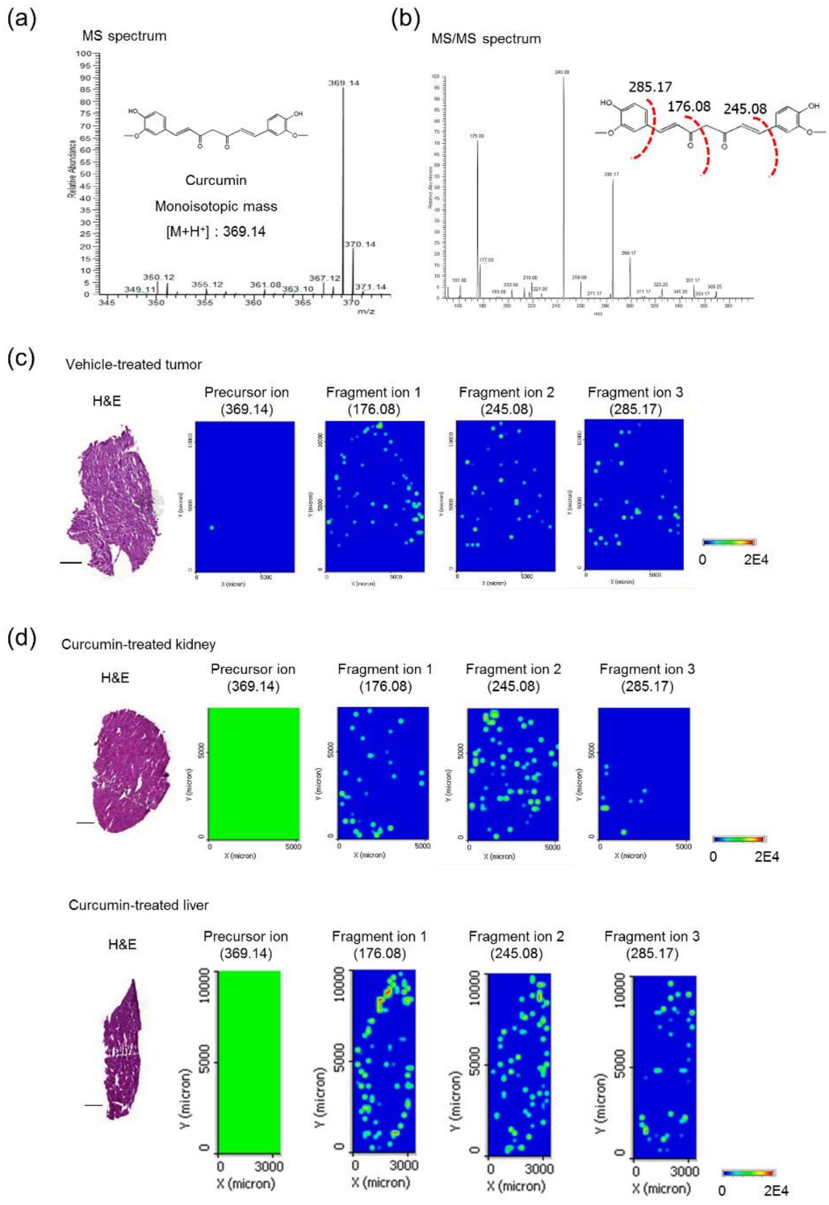


**Figure S1.** Characterization of curcumin by MALDI-MS, MS/MS, and MSI. (a) Full mass spectrum of curcumin. The precursor ion of curcumin (*m/z* 369.14) was obtained in the positive-ion mode by a MALDI LTQ Orbitrap XL mass spectrometer. The images are representative of three independent experiments. (b) MS/MS spectrum of curcumin. CAS structure of precursor curcumin ion (*m/z* 369.14) selected for ionization and identification by MS/MS. Shown here are the fragment ion spectra generated with three principal fragment ions seen at *m/z* 176.08, *m/z* 245.08, and *m/z* 285.17. The image shown is representative of three independent experiments. (c) Comparison of curcumin MSI between vehicle-treated and curcumin-treated tumor tissues. Curcumin MSI (precursor ion, fragment ion 1, fragment ion 2, and fragment ion 3) image from vehicle-treated tumor tissue (normalized to $\alpha$-CHCA matrix, *m/z* = 379.09). These MSI images are representative of three independent experiments. Scale bar, 1 mm. (d) MALDI-MSI images of curcumin (precursor ion, fragment ion 1, fragment ion 2, and fragment ion 3) on curcumin-treated kidney and liver tissues. The results shown are representative of three independent experiments. Scale bar, 1 mm. H&E: hematoxylin and eosin staining.

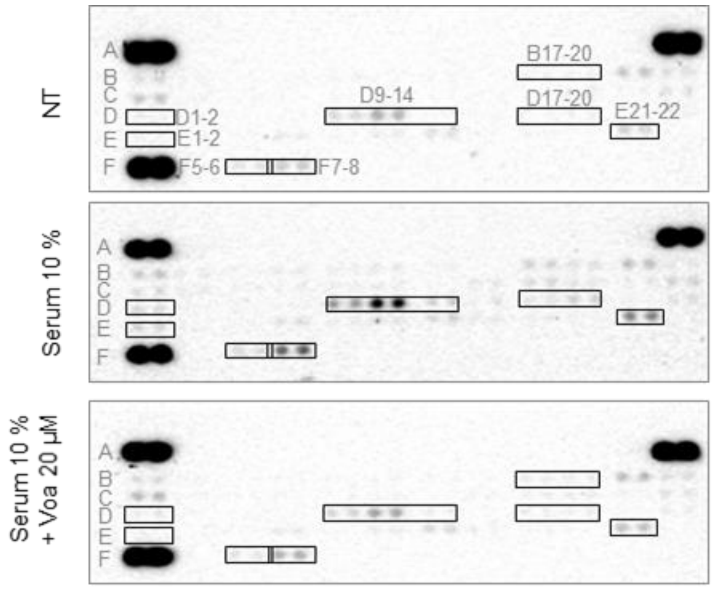


**Figure S2**. Voacangine specifically inhibits VEGFR2 activity. Western blotting result from human phosphoreceptor tyrosine kinases (p-RTKs) array assay in endothelial cells. NT, non-treated control; Voa, voacangine-treated; A1-2, A23-24, and F1-2, reference spots (positive control); F23-24, negative control; B17-20, Insulin receptor; D1-2, Tie-2; D9-10, VEGFR1; D11-12, VEGFR2; D13-14, VEGFR3; D17-18, EphA1; D19-20, EphA2; E1-2, EphA6; E21-22, EphA10; F5-6, EphB3; F7-8, RYK. NT, non-treated control; Voa, voacangine-treated.

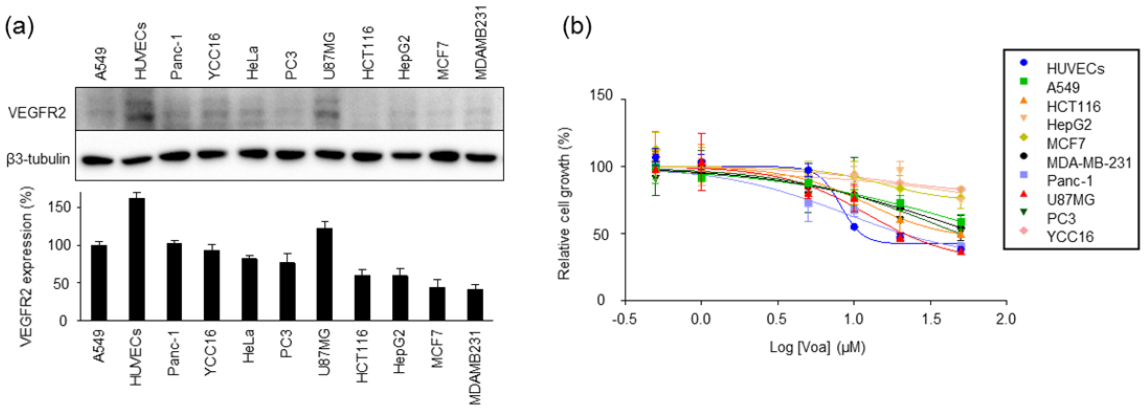


**Figure S3.** Effects of voacangine on tumor growth and angiogenesis related to VEGFR2 expression levels. (a) The expression levels of VEGFR2 in various cell lines. (b) Proliferation assay in indicated cell lines ± voacangine treatment using an MTT assay. Voa, voacangine-treated.

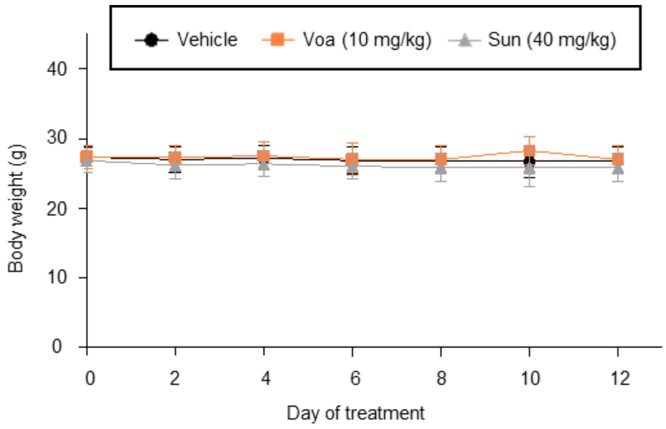


**Figure S4.** Body weight of *in vivo* xenograft model. Body weights of mice were measured. Sun, sunitinib-treated; Voa, voacangine-treated.

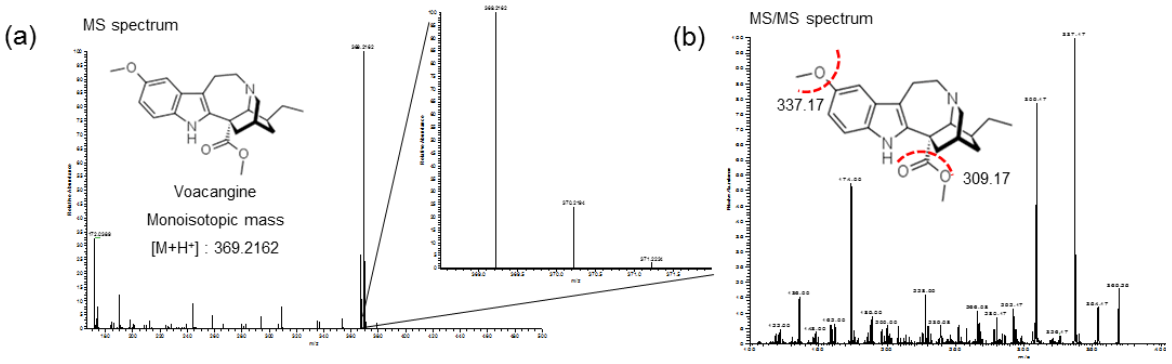


**Figure S5.** Characterization of curcumin by MALDI-MS and MS/MS. (a) The precursor ion of voacangine (*m/z* 369.2162) was obtained in positive-ion mode by a MALDI LTQ Orbitrap XL mass spectrometer. The images are representative of three independent experiments. (b) CAS structure of precursor voacangine ion (*m/z* 369.2162) selected for ionization and identification by MS/MS. Shown here are the fragment ion spectra generated with two principal fragment ions seen at *m/z* 309.17 and 337.17, respectively. The image is representative of three independent experiments.

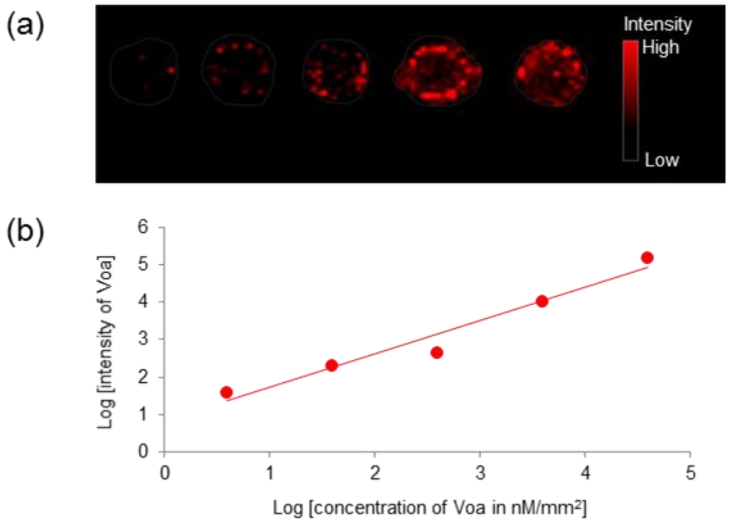


**Figure S6.** Quantitation of a droplet of voacangine on tumor tissue sections. (A) MSI image of MS signal intensities of voacangine (normalized to α-CHCA matrix, m/z = 379.09) at concentrations of 10 nM, 100 nM, 1 μM, 10 μM, and 100 μM, respectively in U87 glioblastoma xenograft tumor tissues. The images are representative of three independent experiments. (B) Calibration curve of voacangine on a logarithmic scale. Equation, y = 0.8901x + 0.831. Correlation Coefficient, R2=0.9571. Voa: voacangine-treated.

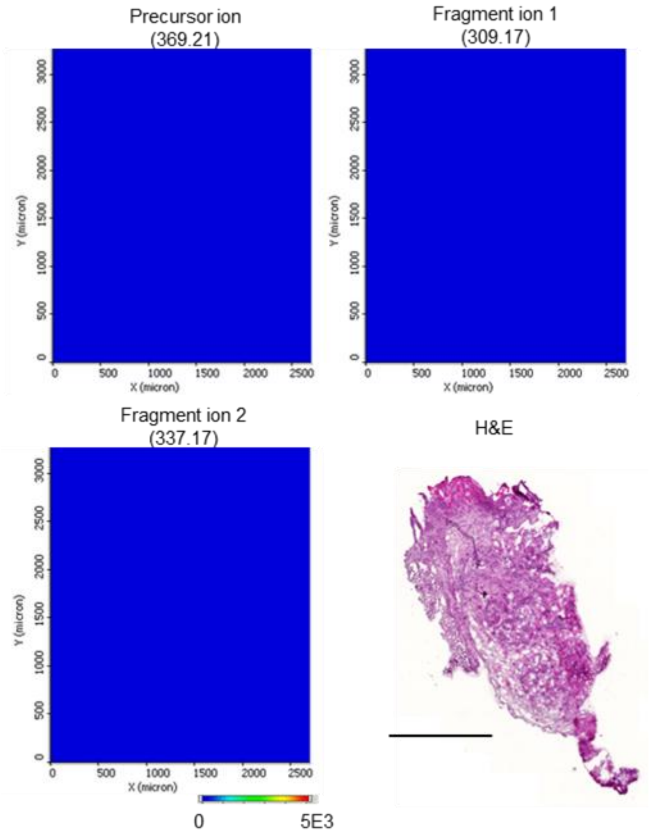


**Figure S7.** Comparison of voacangine MSI between vehicle-treated tumor tissue. Voacangine MSI (precursor ion, fragment ion 1, and fragment ion 2) image from vehicle-treated tumor tissue (normalized to $\alpha$-CHCA matrix, $m/z$ = 379.09). The MSI images are representative of three independent experiments. Scale bar, 1 mm. H&E: hematoxylin and eosin staining.

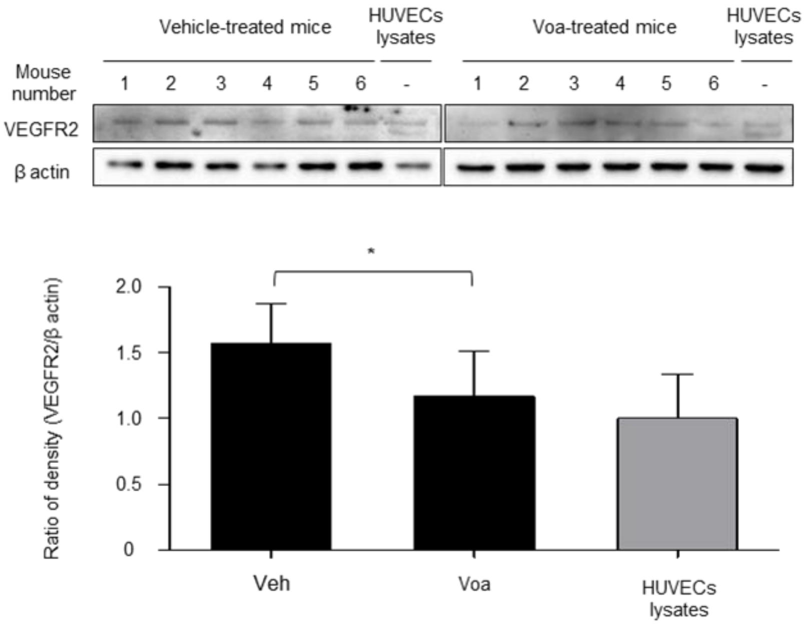


**Figure S8.** Effect of voacangine on the expression of VEGFR2. Effect of voacangine on the expression levels of VEGFR2 in the collected tumor tissues. Immunoblotting analysis of collected tumor tissues obtained from U87 glioblastoma cell xenograft tumors (Vehicle-treated mice, and voacangine-treated mice). These tissues are same tumors from main text Figures 3 and 4. For western blotting tumor sections (10 μm thickness) from tumor tissues were collected and lysed in RIPA buffer. The lysates were immunoblotted with the indicated antibodies. The blots are representative of three independent experiments. Quantitation of the expression levels of VEGFR2. Ratios of density (VEGFR2/β actin) from each group were determined as a ratio relative to the band density from HUVECs lysates by ImageJ. All data from different experimental groups were expressed as the mean ± S.D. * $p < 0.05$ versus HUVECs lysates.

**Table S1**. $IC_{50}$ values of voacangine in study cell lines.

| **$IC_{50}$** | **HUVECs** | **A549** | **HCT116** | **HepG2** | **MCF7** | **MDAMB231** | **Panc-1** | **U87MG** | **PC3** | **YCC16** |
|---|---|---|---|---|---|---|---|---|---|---|
| (μM) | 16.4 | >50 | 49.5 | >50 | >50 | >50 | 18.7 | 19.1 | >50 | >50 |
